# Development and preliminary assessment of the iFIND TBR: all-in- one molecular diagnostic assay for rapid detection of *Mycobacterium tuberculosis* and rifampicin resistance

**DOI:** 10.3389/fcimb.2024.1439099

**Published:** 2024-10-29

**Authors:** Xichao Ou, Zexuan Song, Ruida Xing, Bing Zhao, Shaojun Pei, Chong Teng, Lincai Zhang, Qian Sun, Fang Liu, Hui Xia, Yang Zhou, Yang Zheng, Yuanyuan Song, Zhiguo Zhang, Shengfen Wang, Richard Anthony, Yanlin Zhao

**Affiliations:** ^1^ National Key Laboratory of Intelligent Tracking and Forecasting for Infectious Diseases, National Center for Tuberculosis Control and Prevention, Chinese Center for Disease Control and Prevention, Beijing, China; ^2^ Department of Clinical Laboratory, Children’s Hospital, Capital Institute of Pediatrics, Beijing, China; ^3^ School of Public Health, Peking University, Beijing, China; ^4^ Department of Tuberculosis, Beijing Dongcheng District Center for Disease Control and Prevention, Beijing, China; ^5^ Institute for Tuberculosis Control and Prevention, Gansu Provincial Center for Disease Control and Prevention, Lanzhou, China; ^6^ Department of Clinical Laboratory, Tuberculosis Dispensary of Changping District, Beijing, China; ^7^ National Institute for Public Health and the Environment, Bilthoven, Netherlands

**Keywords:** tuberculosis, *Mycobacterium tuberculosis*, the iFIND TBR, rifampicin resistance, rapid detection

## Abstract

**Introduction:**

Early and accurate diagnosis of tuberculosis (TB) is crucial for initiating timely treatment and preventing new infections. In this study, we introduced the iFIND TBR assay, an automated all-in-one tuberculosis detection approach that simultaneously detect *Mycobacterium tuberculosis* (MTB) and rifampicin (RIF) resistance.

**Methods:**

The limits of detection (LOD), sensitivity, specificity, and RIF-R rpoB mutation detection of the iFIND TBR were tested on Mycobacterium tuberculosis DNA or sputum samples spiked with known numbers of M.tuberculosis H37Rv. Frozen clinical samples from patients suspected of having TB were also tested.

**Results:**

The LOD of the iFIND TBR for MTB detection were 13.34 CFU/ml (95% CI, 11.71-16.47), and for RIF resistance was 109.79CFU/mL (95% CI, 95-138.19). The iFIND TBR assay accurately distinguish MTB strains from non-tuberculous mycobacteria (NTM) without any cross reactivity. Testing on 157 clinical sputum samples, compared with the bacteriologically TB standard, the overall sensitivity and specificity of the iFIND TBR was 100% (95%CI, 94.64, 100) and 85.29% (95% CI, 74.61, 92.72), respectively. When assessing RIF susceptibility, the iFIND TBR achieved a sensitivity of 98.15% (95% CI, 90.11–99.95) and a specificity of 85.71% (95% CI, 67.33–95.97), compared with phenotypic drug susceptibility testing. Discordant RIF susceptibility results were more frequently observed in samples exhibiting heteroresistance.

**Discussion:**

These findings demonstrate that iFIND TBR assay performs well in detecting TB and RIF resistance, and shows promise as a point-of-care tool in resource-limited areas.

## Introduction

Tuberculosis (TB), caused by *Mycobacterium tuberculosis* (MTB), remains a major global public health challenge. The WHO Global Tuberculosis Report of 2023 estimated 10.6 million people fell ill and 1.3 million deaths with TB in 2022 (WHO, 2023). Timely and accurate TB diagnosis is a crucial element of the worldwide strategy to eradicate tuberculosis (Consortium et al., 2022).

Currently, clinical radiography and smear microscopy are commonly employed in the diagnosis of TB, but microscopies limited sensitivity and inability to distinguishing non-tuberculous mycobacteria (NTM) from MTB limits diagnostic utility (Steingart et al., 2006). Although culture remains the “gold standard” for TB diagnosis, its application is limited by the biosafety infrastructure required and need for lengthy cultures (2–8 weeks) (Schumacher et al., 2019). Recent advancements in molecular diagnostics have significantly accelerated the development of rapid TB testing methods with good performance characteristics (Kontsevaya et al., 2023), particularly the Xpert MTB/RIF (Cepheid, USA). It is an automated, cartridge-based system, which employs a semi-nested real-time polymerase chain reaction (PCR) technique to specifically target the *rpoB* gene, enabling the simultaneous detection of MTB and rifampicin resistance within 2 h (Helb et al., 2010). The WHO has endorsed the Xpert MTB/RIF and the next- generation Xpert Ultra test as the initial diagnostic test for all individuals presumed to have pulmonary TB (Xpert MTB/RIF Implementation Manual: Technical and Operational ‘How-To’; Practical Considerations, 2014 2014). Nevertheless, the relatively high cost limits the accessibility of Cepheid Xpert testing, especially in less-developed countries. Several novel diagnostic approaches for TB diagnosis have been successfully developed (Kontsevaya et al., 2023), such as CRISPR-Cas-based diagnostic assays, but these applications are still in their infancy (Huang et al., 2023). Thus, the development of lower cost and highly sensitive molecular diagnostics assays is needed.

Recently, IFIND Company (Beijing, China) developed the iFIND TBR assay, an all-in-one tuberculosis detection assay for identification and rifampin susceptibility testing. The iFIND TBR assay integrates sample processing and detection into a single-use cartridge. It uses five molecular beacons targeted to the rifampicin resistance determining region (RRDR) of *rpoB* gene to detect rifampin resistance. Two additional probes targeting the MTB multicopy IS6110 and IS1081 genes are used to identify the presence of MTB. Herein, we describe in detail the design of the cartridge and methodology, as well as practical applications for TB detection and identification of rifampicin resistance testing.

## Methods

### Assay components

**Figure 1:**
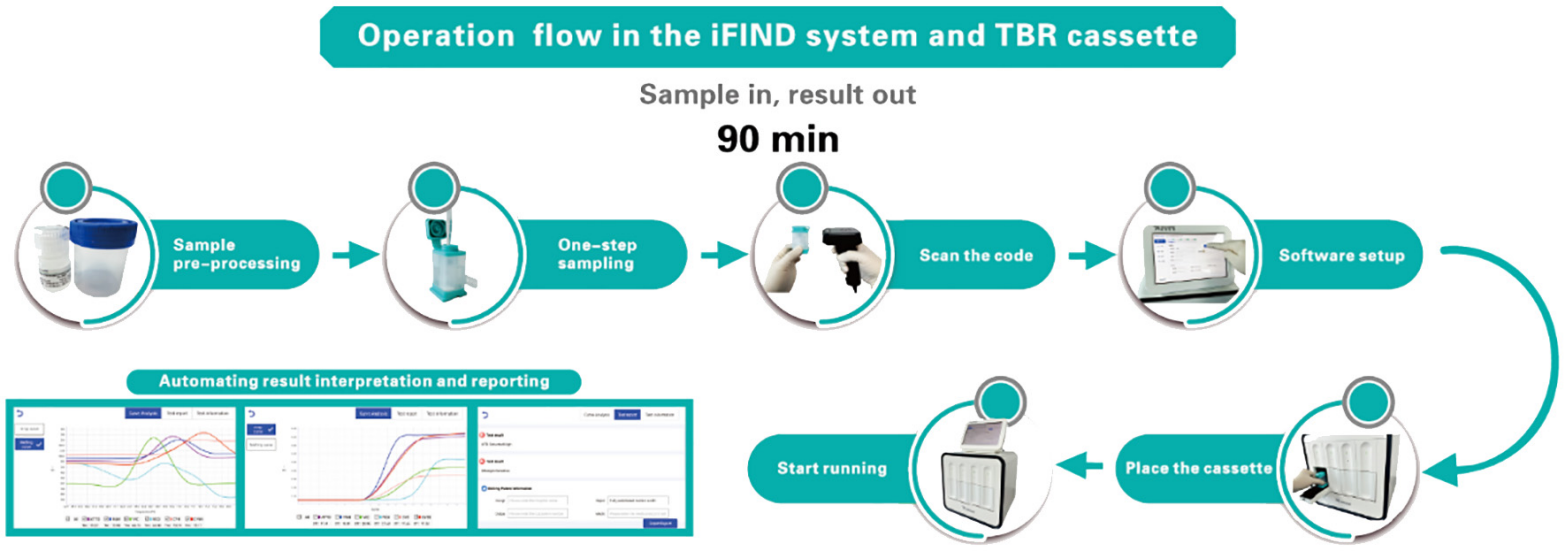
Schematic of the iFIND workflow.

The iFIND TBR assay is based on the iFIND automated system, a fully integrated, automated, and all-in-one nucleic acid testing and analysis system (**Figure 1**). The iFIND system comprises two primary components: (i) The disposable microfluidic cassette, which carries the entire process of reaction between the sample and the nucleic acid reagent. This cassette utilizes one-piece injection molding technology, significantly reducing production costs and preventing aerosol contamination at the source, which not only ensures the accuracy of the results but also makes it more it more feasible for widespread adoption. (ii) The iFIND instrument utilizes microfluidic control and precision injection molding technology, integrating sample enrichment, extraction, amplification, and analysis to realize fully automated “sample in, result out”. The temperature control technology of iFIND is based on the combination of self-developed ultra-fast temperature control technology, incorporating a Peltier effect semiconductor temperature control element, which ensures the fast-warming rate and greatly improves the cooling rate of the reaction zone, realizing a maximum warming rate of ≥11.5°C/s (50°C to 95°C) and maximum cooling rate of ≥10°C/s (95°C to 50°C). Improvements in temperature ramp speed significantly reduce the time needed to run the assay, with iFIND nearly 20% faster than comparable tests. Additionally, the iFIND TBR assay instrument is only suitable for indoor use, where the indoor ventilation must be excellent, and no corrosive gas should be present. The operating ambient temperature should be maintained between 10°C and 30°C, and the relative humidity must be ≤70%. The operating atmospheric pressure should be within the normal range, suitable for altitudes below 2,000 m. Furthermore, the iFIND TBR device requires regular maintenance to ensure its optimal performance and longevity.

The iFIND TBR assay was designed to amplify insertion sequences IS6110, IS1081, and *rpoB* genes specific to members of the *Mycobacterium tuberculosis* complex, and mutations within the rifampin resistance-determining region (RRDR) of the *rpoB* gene. Primers targeting *M. tuberculosis rpoB* in a hemi-nested PCR and five rpoB-specific molecular beacons were optimized to minimize cross-amplification and to maximize mutation detection.

A semi-nested molecular beacon PCR to detect Bacillus cereus DNA was also included as an internal control for the sample processing and PCR steps. All six molecular beacons are multiplexed within the same reaction. The iFIND instrument plots real-time amplification curves and melting curves based on the detected signals, thus enabling the quantitative detection in samples.

### The iFIND TBR procedure

The sputum specimen is placed in a sterile sputum box, the volume of sample treatment solution (including NaOH and other chemical substances) is added one to two times depending on the viscosity of the sputum specimen, and the lid on is screwed tightly, vortexed, oscillated for approximately 1 min to homogenize the sample, and then incubated at room temperature for 15 min until the specimen is completely liquefied. 2 to 3 ml of digested sputum is transferred to the iFIND TBR cassette, then the lid is closed, and the cassette is loaded into the iFIND instrument. Subsequent steps will be performed automatically by the instrument. The iFIND instrument is opened, and the automated detection scheme is selected corresponding to TBR in the software. “Open” on the operation page is clicked, the cartridge is gently placed into the slot of the corresponding detection unit of the iFIND instrument, the door of the detection chamber is closed, and “Start Run” is clicked to carry out the detection. All the test data will be automatically interpreted by the software, and the final output of TB identification and rifampicin resistance results are displayed.

### Hetero-resistance detection

To test the capacity of iFIND TBR to detect hetero-resistance, mutant DNA was added to wild-type DNA at various percentages. Briefly, the *rpoB* (rifampicin resistance determining region) RRDR of DNA samples obtained from different clinical strains was confirmed for the presence or absence of mutations by Sanger sequencing and quantified using a NanoDrop Microvolume Spectrophotometer (Thermo Fisher Scientific, Wilmington, DE, USA). Mixtures of wild-type DNA and DNA with different RRDR mutations were prepared by combining equal concentrations of both at different ratios, ranging from 90% mutant DNA to 0.5% mutant DNA in the mixture. These mixed samples were then used to determine the minimum proportion of mutant DNA required to produce a rifampicin-resistant (RIF-R) result in the assay.

### Sensitivity and specificity evaluation of the iFIND TBR

The analytical sensitivity and limit of detection (LOD) of the iFIND TBR assay were determined by spiking *M. tuberculosis* H37Rv (ATCC 27294) into *M. tuberculosis*-negative sputum and testing each sample. The CFU stock preparation and sputum spiking protocols were accordance with a standard protocol as previously described (Chakravorty et al., 2017). Attenuated strains of *M. tuberculosis* H37Rv were cultured by inoculating an optical density 600 nm (OD600) culture 1:100 in 10 ml of 7H9 broth supplemented with 10% Middlebrook OADC Growth supplement and 0.05% Tween 80 (Sigma- Aldrich, St Louis, MO). Both of the strains were then grown to an optical density OD600 of 0.6–0.8 and subcultured one additional time by making a 1 to 100 dilution into fresh growth media and reincubating the culture until the OD600 again reached a density of 0.6–0.8. The culture stock was mixed three times using a serological pipette and then divided into 200-µl aliquots and stored at −80°C. An additional 1- ml aliquot of the stock culture was sonicated for 30 s using a Branson CPX1800 Ultrasonic water bath (Branson, Danbury, CT, USA) and rested for 30 s on ice. This was repeated twice more, and then the culture was placed on ice for 6 min. Tenfold serial dilutions were then performed by adding 400 µl of the sonicated culture to 3.6 ml of supplemented media; subsequent dilutions were performed in the same manner except that each dilution was mixed by pipetting 10 times, aspirating from the bottom of the tube and releasing on the top, and then 10 times aspirating from the top and releasing on the bottom. Culture plates were checked for growth at 1 week after plating and then on alternate days after that. Colony counts were performed once colonies were clearly visible (at approximately 2 to 3 weeks). Colony counts ranging between 10 and 300 were included for estimation of the CFU/ml in the respective cultures.

The analytical sensitivity experiments were performed by spiking *M. tuberculosis* H37Rv CFU in sputum with the final concentrations ranging from 0 CFU/ml to 25 CFU/ml for TB detection and 20 CFU/ml to 140 CFU/ml for RIF susceptibility analysis. Each dilution was tested 20 times. The assay LOD was determined by calculating the minimum CFU required to detect MTB in at least 95% of test runs. Additionally, to determine the analytical specificity and cross-reactivity of the iFIND TBR assay, the DNA of common non-tuberculous mycobacteria (NTM) and other bacterial microflora present in sputum and the upper respiratory tract were also added in each sample. Each test was repeated three times.

### The clinical sputum samples

The performance of iFIND TBR on clinical sputum samples was assessed in a small-scale retrospective accuracy investigation at the NTRL in China. Sputum samples were collected from 173 suspected pulmonary tuberculosis patients from the Tuberculosis Dispensary of Changping District. The sputum samples were homogenized for 1 min with a vortex and glass beads and divided, with some aliquots frozen at −80°C for later analysis by iFIND TBR assay and Xpert MTB/RIF assay, the remainder subjected to quantitative culture on the BACTEC MGIT 960 liquid culture system (Becton, Dickinson Diagnostic System, NJ, USA). The critical concentration of rifampicin susceptibility used in this study is 0.5 mg/L, in accordance with the WHO guidelines (Yu et al., 2023).

### The MTB samples and DNA extraction

A total of 143 MTB strains were randomly selected from the National Tuberculosis Reference Laboratory (NTRL) in China. Genomic DNA of all the strains were extracted and purified using CTAB according to the previous report. All extracted DNA samples stored at −20°C.

### Statistical analysis

All statistical analysis were performed by SPSS v18.0 software (SPSS Inc., Chicago, Illinois). Lower and upper 95% confidence intervals (95% CIs) were created for the curve after binary logistic regression findings were fitted through the tested concentrations. The point at which the 95% probability level crossed the upper and lower 95% CIs, indicating the LOD, was used to calculate the 95% confidence interval for the minimal input concentration. Sensitivity and specificity values of iFIND TBR were described as point estimates and 95% confidence intervals. The *P*-value less than 0.05 was considered statistically significant.

## Results

### RIF resistance detection of the iFIND TBR

**Figure 2:**
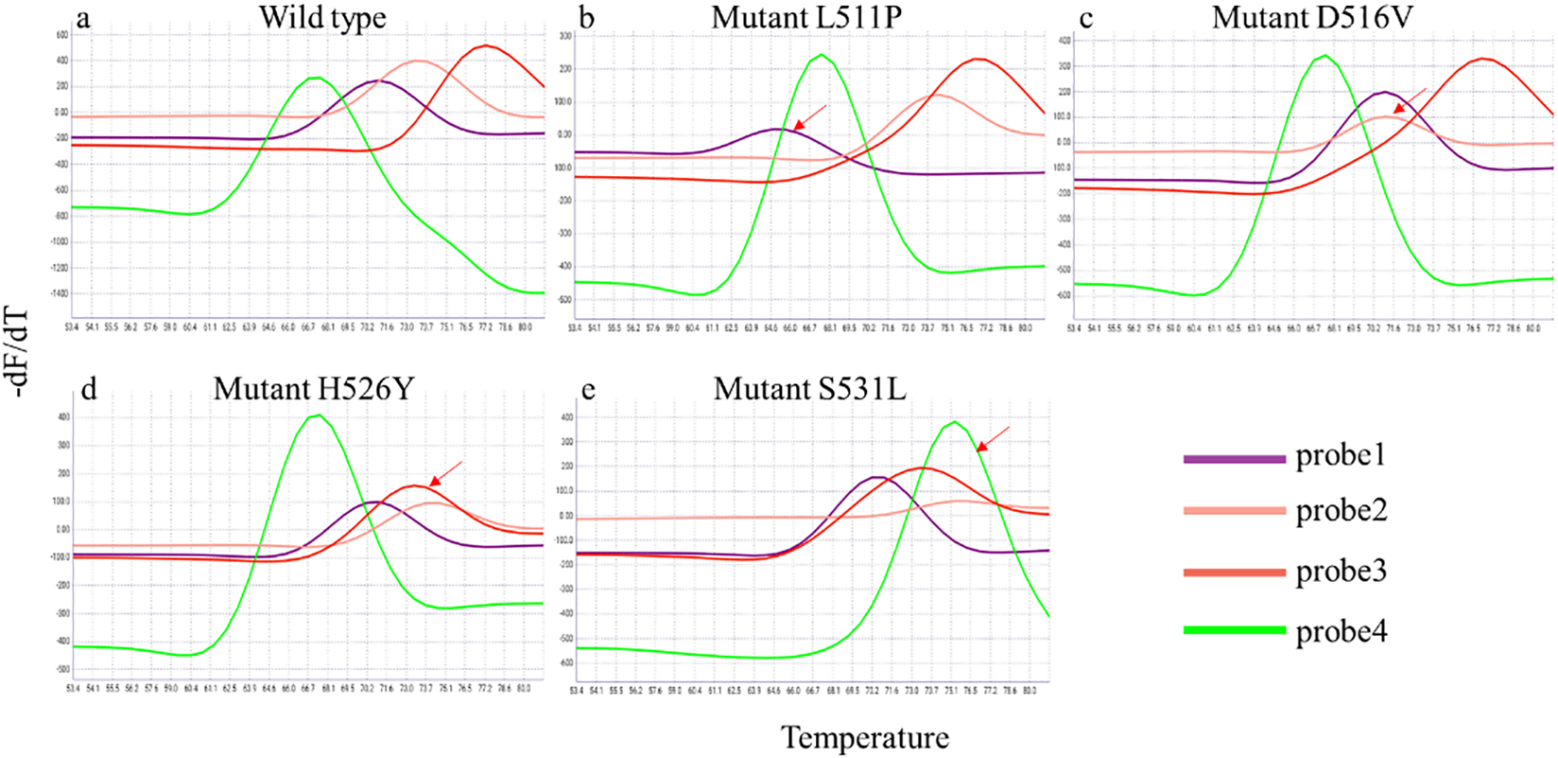
Detection of rpoB gene mutations associated with RIF resistance in the iFIND TBR. The peaks for wild-type M. tuberculosis **(A)** identify a test sample as rifampin susceptible. The shift in one or more of the peaks away from the wild type’s melting temperature **(B–E)**, identify a sample as a *rpoB* mutant and rifampin resistant. The shift in the melt peak is indicated by arrows.

In this study, four fluorescent probes targeting the *rpoB* gene to detect the RIF resistance, and all mutations resulted in a reproducible and measurable shift in melting Tm peaks by one or more of the *rpoB* SMB probes (**Figure 2**). We evaluated the RIF resistance detection of iFIND TBR assay with nucleic acids of 143 MTB strains. These strains contain 71 genotypic RIF resistant, and the mutation panel included double and triple mutations, deletions, and stop codons (**Supplementary Table S1**). All the strains harboring RIF- associated resistance mutation in RRDR were reproducibly identified by the iFIND TBR software as “RIF resistance detected”. One strain carried the mutation *rpoB*_V170F which was not detected, due to the fact that this mutation lies outside the detection range of the iFIND TBR method. These results demonstrated that iFIND TBR assay can be used for the detection of RIF resistance of MTB strains.

### Sensitivity of the iFIND TBR

**Figure 3:**
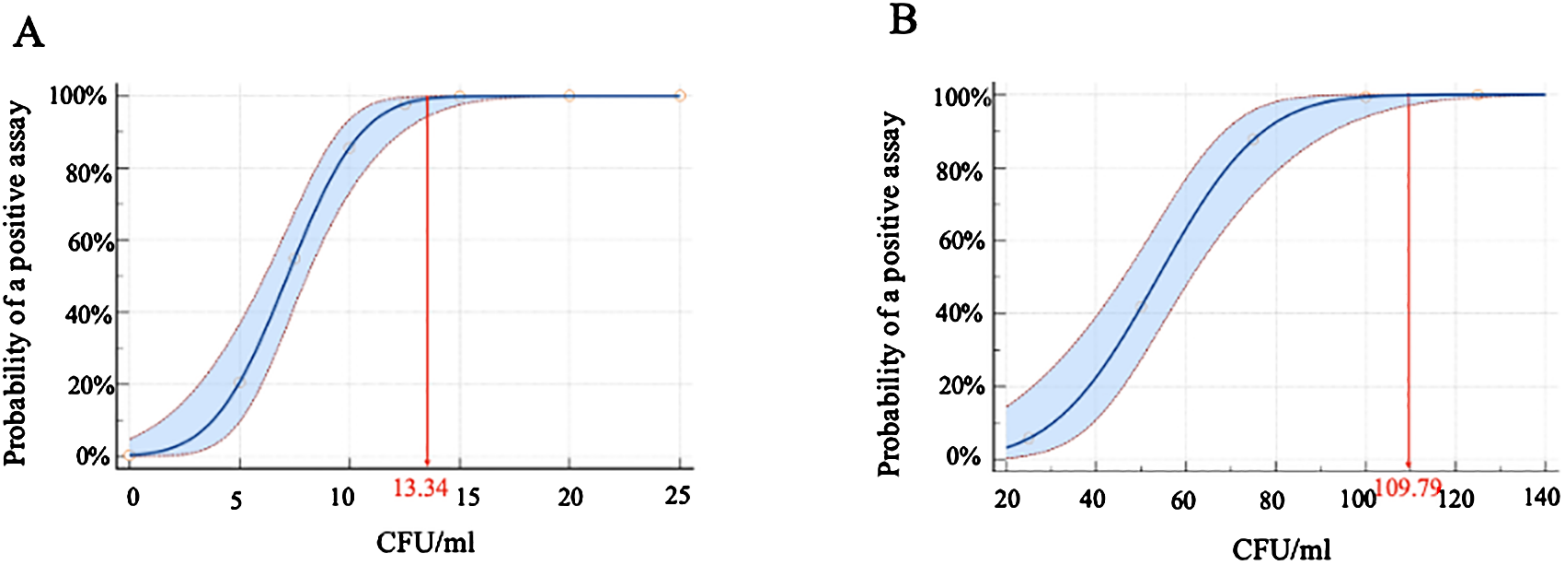
The limit of detection (LOD) for *M. tuberculosis* H37Rv by iFIND TBR. **(A)** The limit of detection of tuberculosis detection. **(B)** The limit of detection of rifampin susceptibility.

The limit of detection (LOD) of the iFIND TBR assay was evaluated by spiking serial dilutions of quantified CFU into sputum samples. The result showed that the iFIND TBR assay could detect the presence of MTB correctly in all dilutions containing 25 CFU/ml or more. The calculated TB detection LOD for iFIND TBR was 13.34 CFU/ml (95% CI, 11.71–16.47) (**Figure 3A**), which represents an approximately 10-fold improvement over that of Xpert, as reported (131 CFU/ml) (Helb et al., 2010). Additionally, the calculated LOD for RIF susceptibility was 109.79 CFU/ml (95% CI, 95–138.19 CFU/ml), based on the fact that RIF susceptibility detected correctly 100% by iFIND TBR at 100 CFU/ml (**Figure 3B**).

### Heteroresistance detection of the iFIND TBR

**Figure 4:**
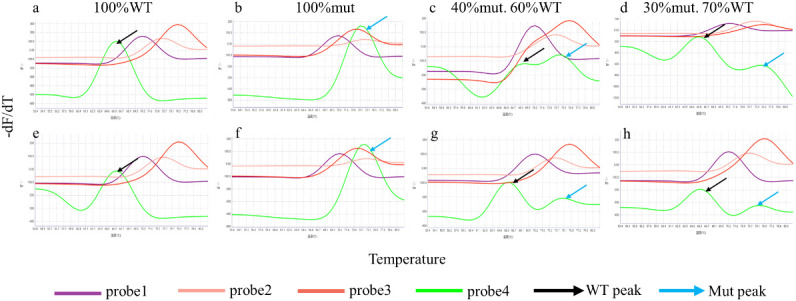
Heteroresistance detection of the iFIND TBR. Samples containing 500 CFU/ml **(A–D)** and 250 CFU/ml **(E–H)** of *M. tuberculosis* DNA were created using different proportions of wild-type and rifampin-resistant *rpoB* S531L mutant DNA.

The iFIND TBR assay demonstrated the ability to detect heteroresistance using testing mixtures of wild-type DNA and DNA containing the rpoB S531L mutation, which is the most common mutation associated with rifampicin resistance (The, 2022). The results showed that the rpo4 probe has a distinct double peak in a proportion of mixtures containing as little as 30% mutant DNA (**Figure 4**; **Supplementary Figure S1**). Similar results were observed at both 500 CFU/ml and 250 CFU/ml concentrations.

### Specificity of the iFIND TBR

The specificity of the iFIND TBR assay was tested on genomic DNA templates of 15 bacteria, including 9 different isolates of non-tuberculous mycobacteria (NTM) and 6 different Gram-positive and -negative bacteria (**Supplementary Table S2**). The two MTB detection probes, targeting the IS6110 and IS1081 genes, yielded no signals, resulting in an output of “MTB not detected” for all tested replicates. Thus, no cross-reactions were observed from non-MTB strains. These data suggest that the iFIND TBR assay was highly specific for TB detection.

### Clinical feasibility validation of iFIND TBR

A total of 173 unique sputum samples were collected and 16 samples were excluded because culture failed or was contaminated. Thus, we performed a limited clinical validation of the performances of iFIND TBR using 157 sputum samples (**Supplementary Figure S2**). Compared with culture results, the overall sensitivity and specificity of iFIND TBR for TB detection were 100% (95% CI, 95.94, 100) and 85.29% (95% CI, 74.61, 92.72), respectively, and those of Xpert were 98.88% (95% CI, 93.90, 99.97) and 89.71% (95% CI, 79.93, 95.76), respectively (**Table 1**). However, some discrepancies were observed between the results of the iFIND TBR, Xpert, and culture method (**Supplementary Table S3**).

**Table 1:** Diagnostic performance of the iFIND TBR and Xpert assay for TB using culture.

	% sensitivity (95% CI)	% specificity (95% CI)	PPV	NPV	Kappa
TBR	100 (95.94, 100)	85.29 (74.61,92.72)	89.9 (82.21~95.05)	100 (93.84~100)	0.8680 (0.7895~0.9465)
Xpert	98.88 (93.90,99.97)	89.71 (79.93,95.76)	92.63 (85.41~96.99)	98.39 (91.34~99.96)	0.8951 (0.8246~0.9657)

In this study, 88 samples were detected MTB by iFIND TBR, Xpert, and phenotypic drug susceptibility. Six samples without phenotypic drug susceptibility were excluded, totaling 82 clinical samples analyzed. The results of iFIND TBR and Xpert were compared with the results of RIF susceptibility. Compared with phenotypic drug susceptibility for rifampin susceptibility testing, the sensitivity and specificity of iFIND TBR for the detection of RIF susceptibility were 98.15% (95% CI, 90.11, 99.95) and 85.71% (95% CI, 67.33, 95.97), respectively, whereas those of Xpert were 94.44% (95% CI, 84.61, 98.84) and 92.86% (95% CI, 76.50, 99.12), respectively (**Table 2**). In addition, four samples were detected as RIF resistant by the MGIT method, but susceptible by iFIND TBR (**Supplementary Table S4**). Upon Sanger sequencing (**Supplementary Figure S2**), two samples were shown to hetero-resistant, with a mixture of the wild type and S531L and D516V mutants, respectively, which were detected as resistant by RIF susceptibility test but missed by iFIND TBR. The other two samples showed no mutations in RRDR, which could be the result of resistance due to other mutations outside of RRDR (Huo et al., 2020). One sample showed the mixture of the wild type and H526Y mutant; it was detected to be RIF resistant by iFIND TBR but susceptible by the MGIT method.

**Table 2:** Diagnostic accuracy of the iFIND TBR and Xpert assay for RIF susceptibility.

	% sensitivity (95% CI)	% specificity (95% CI)	PPV	NPV	Kappa
TBR	98.15 (90.11, 99.95)	85.71 (67.33,95.97)	92.98 (83.00~98.06)	96.00 (79.65~99.90)	0.8680 (0.7431~0.9785)
Xpert	94.44 (84.61,98.84)	92.86 (76.50,99.12)	96.23 (87.02~99.54)	89.66 (72.65~97.81)	0.8656 (0.7515~0.9796)

## Discussion

Novel molecular diagnostics with enhanced sensitivity are urgently needed to facilitate the diagnosis of tuberculosis and guide therapeutic decisions (Pai et al., 2023). In this study, we have developed a novel molecular diagnostic assay, named iFIND TBR, with good TB detection capabilities and more definitive identification of RIF resistance.

Our data showed that the LOD of iFIND TBR assay is 13.34 CFU/ml using sputum samples spiked with known numbers of MTB CFU. This represents a 10-fold enhancement in analytical sensitivity compared with the Xpert assay (131 CFU/ml) (Helb et al., 2010), and is in line with that of the Xpert Ultra, the most sensitive assay currently available, at 15.6 CFU/ml (Chakravorty et al., 2017). This improvement in sensitivity can be attributed to the utilization of multicopy genes, as opposed to single-copy genes. Specifically, the iFIND TBR assay targets the IS6110 and IS1081 genes, both of which are multicopy genes in MTB. Additionally, including the IS1081 gene enhances the assay’s ability to detect rare MTB strains that completely lack the IS6110 gene (Lok et al., 2002).

The iFIND TBR assay also facilitates detection of RIF resistance mutations within RRDR. Previous studies showed that rifampicin- resistant mutations showed a clear predominance of well-established mutations in RRDR of the *rpoB* gene (He et al., 2022; Song et al., 2023). In this study, our data confirmed that the iFIND TBR method is effective in detecting mutations in the RRDR region, crucial for diagnosing clinical rifampicin resistance. However, there are some samples with discordant results between iFIND TBR and phenotypic DST, but consistent with the Xpert assay. These conflicting results might be due to hetero-resistance and in one case a resistance-associated mutation outside the RRDR region, such as *rpoB*_V170F (Walker et al., 2022). In addition, samples with low bacterial load are known to sometimes result in inconsistent molecular RIF susceptibility results. For example, Deng et al. observed that discrepancies in RIF susceptibility results between InnowaveDX and Xpert occurred more frequently in samples with a very low bacterial load (Deng et al., 2023). Huo et al. have also demonstrated that 20% isolates exhibiting discordant results between Xpert and phenotypic DST were devoid of *rpoB* mutations, predominantly observed in specimens with very low bacterial loads (Huo et al., 2020). Thus, caution is advised when interpreting rifampicin susceptibility results in cases with low bacterial loads.

Despite over a decade of utilization and substantial public and philanthropic investments totaling billions of dollars, products like Xpert MTB/RIF (GeneXpert) remain prohibitively expensive and challenging to access and maintain in countries with high TB burden and developing nations (Lawn et al., 2013; Pai et al., 2023). Compared with Xpert, the iFIND TBR assay has a low cost per test and a better TB detection capability. Additionally, the iFIND TBR assay from sample preprocessing to result readout can be completed within 90 min, which greatly improves the efficiency of the test. Limited studies on frozen clinical samples have shown that the iFIND TBR has high sensitivity and specificity for clinical use. These advantages further demonstrate the potential of the iFIND TBR method for application in resource-constrained settings, suggesting the iFIND TBR as an alternative and affordable option being routinely practiced.

There were several limitations in this study. Firstly, there were fewer strains with inconsistent TBR and phenotypic DST, and whether this was due to low bacterial loads has not been verified. Secondly, we did not compare the diagnostic accuracy of iFIND TBR with the Xpert Ultra in clinical practice, which had a better TB detection capability (Chakravorty et al., 2017). In addition, this technology is limited by the number of mutations that can be probed.

## Conclusion

In this study, we have evaluated a novel molecular diagnostic method, named iFIND TBR, which shows promising potential in accurately detecting tuberculosis and determining rifampicin susceptibility. The whole test procedure for iFIND TBR was simple to perform, can be completed within 90 min, and provides the advantages of high specificity, sensitivity, and rapid results. This all-in-one approach has the potential as a simple, scalable, and time-saving alternative tuberculosis and rifampicin resistance detection.

## Data Availability

The datasets presented in this study can be found in online repositories. The names of the repository/repositories and accession number(s) can be found in the article/**Supplementary Material**.
