# Supplementary material for: Development and preliminary assessment of the iFIND TBR: all-in- one molecular diagnostic assay for rapid detection of Mycobacterium tuberculosis and rifampicin resistance
Source: Front Cell Infect Microbiol. 2024 Oct 29;14:1439099. doi: 10.3389/fcimb.2024.1439099 (PMC11554655; doi:10.3389/fcimb.2024.1439099)
Supplement: Supplementary file 1 [file Table1.docx]

Table S1. The RIF resistance detection of iFIND-TB with nucleic acids of MTB strains.

| Number | Mutation | iFIND-TB |
| --- | --- | --- |
| 3 | rpoB_L452P; | R |
| 32 | rpoB_S450L; | R |
| 1 | rpoB_K446T; | R |
| 2 | rpoB_L430P; | R |
| 1 | rpoB_Q432P; | R |
| 2 | rpoB_S441L | R |
| 1 | rpoB_S450T; | R |
| 3 | rpoB_H445T | R |
| 1 | rpoB_H445S | R |
| 4 | rpoB_H445A | R |
| 1 | rpoB_A435T | R |
| 2 | rpoB_H445L | R |
| 1 | rpoB_G432H | R |
| 1 | rpoB_A435A + rpoB_L430P | R |
| 1 | rpoB_H445G + rpoB_L430P | R |
| 1 | rpoB_H445A + rpoB_S441L | R |
| 1 | rpoB_I480V + rpoB_S450L | R |
| 1 | rpoB_L449M + rpoB_S450P | R |
| 1 | rpoB_A435V + rpoB_P+454S | R |
| 1 | rpoB_D435Y + rpoB_Q429H; | R |
| 1 | rpoB_L443S + rpoB_S450L; | R |
| 1 | rpoB_D435Y + rpoB_L430P; | R |
| 1 | rpoB_V170F; | S |
| 1 | rpoB_1326_del_1_GT_G; rpoB_1333_del_3_CACA_C; rpoB_1335_del_3_CAAG_C; rpoB_L443W; rpoB_T444P; | R |
| 1 | rpoB_1333_del_3_CACA_C; rpoB_L443W; rpoB_T444P; | R |
| 5 | rpoB_c.1297_1305delTTCATGGAC | R |
| 72 | WT | S |

Table S2. Bacterium species used in this study.

| Pathogens | Source of strains | No. of  strains | Results of iFIND TBR |
| --- | --- | --- | --- |
| *Mycobacterium tuberculosis* | ATCC 27294 | 1 | P |
| *Mycobacterium avium subsp. avium* | ATCC 25291 | 1 | N |
| *Mycobacterium fortuitum subsp. fortuitum* | ATCC 6481 | 1 | N |
| *Mycobacterium phlei* | ATCC 11758 | 1 | N |
| *Mycobacterium terrae* | ATCC 19619 | 1 | N |
| *Mycobacterium szulgai* | Isolated strains (CDC) | 1 | N |
| *Mycobacterium kansasii* | ATCC 12478 | 1 | N |
| *Mycobacterium asiaticum* | Isolated strains (CDC) | 1 | N |
| *Mycobacterium scrofulaceum* | ATCC 19981 | 1 | N |
| *Mycobacterium gordonae* | ATCC 14470 | 1 | N |
| *Mycobacterium abscessus* | ATCC 19977 | 1 | N |
| *Nocardia brasiliensis* | Isolated strains (CDC) | 1 | N |
| *Corynebacterium* | Isolated strains (CDC) | 1 | N |
| *Streptococcus pneumoniae* | Isolated strains (CDC) | 1 | N |
| *Legionella pneumophila subsp pneumophila* | Isolated strains (CDC) | 1 | N |
| *Bordetella pertussis* | Isolated strains (CDC) | 1 | N |

^a^CDC, Chinese center of disease control and prevention

Table S3. The Inconsistent results of iFIND TBR, Xpert and MTB Culture for TB detection.

| No. | iFIND TBR | Xpert | MTB Culture | Sequencing Result | Clinical Diagnosis |
| --- | --- | --- | --- | --- | --- |
| 1 | P | P | N | P | None |
| 2 | P | P | N | P | None |
| 3 | P | P | N | P | None |
| 4 | P | P | N | P | None |
| 5 | P | P | N | P | None |
| 6 | P | P | N | P | None |
| 7 | P | P | N | P | None |
| 8 | P | N | N | P | P |
| 9 | P | N | N | P | P |
| 10 | P | N | N | P | P |

(P: MTB DETECTED; N: MTB NOT DETECTED; None: Null Results)

Table S4. The Inconsistent results of iFIND TBR, Xpert and phenotypic drug susceptibility testing for RIF detection.

| No. | iFIND TBR | Xpert | Drug Susceptibility Test | Sequencing Result |
| --- | --- | --- | --- | --- |
| 1 | S | S | R | WT |
| 2 | S | S | R | WT |
| 3 | S | R | R | MIX (D516V) |
| 4 | R | R | S | MIX (H526Y) |
| 5 | S | R | R | MIX (S531L) |

R: RIF Resistant; S: RIF Susceptible; WT: wild type; MIX: mixtures of wild-type DNA and DNA containing the *rpoB* mutation

Figure S1. Heteroresistance detection of the iFIND TBR. Samples containing 500 CFU/ml (a,b) and 250 CFU/ml (c,d) of *M. tuberculosis* DNA were created using different proportions of wild-type and rifampin-resistant *rpoB* S531L mutant DNA.


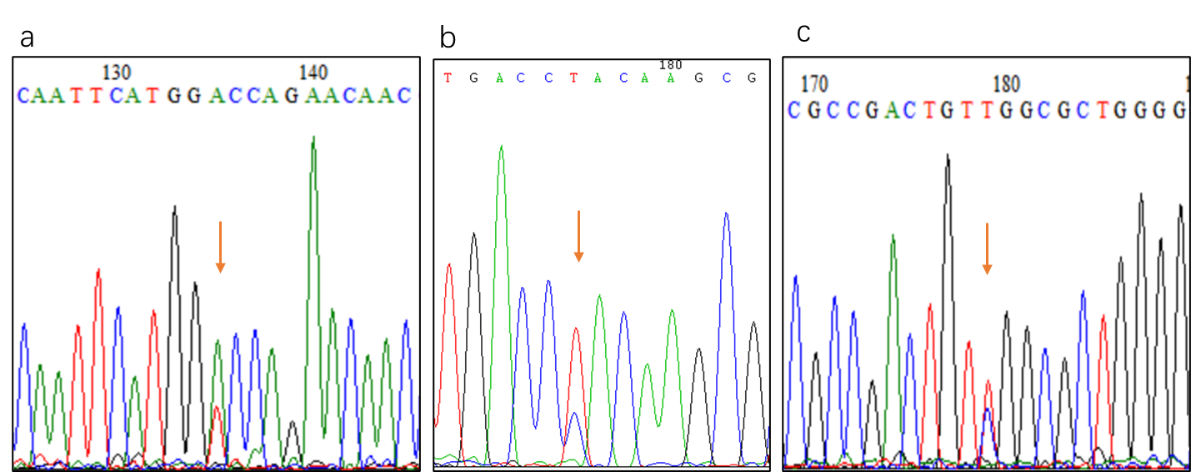


Figure S2. The Sequencing Results of the Inconsistent results of Drug Susceptibility Testing for RIF. (a). Mixtures of wild-type DNA and DNA containing the *rpoB* D516V mutant. (b). Mixtures of wild-type DNA and DNA containing the *rpoB* H526Y mutant. (c) Mixtures of wild-type DNA and DNA containing the *rpoB* S531L mutant.
